# Impact of fiberoptic bronchoscopy with bronchoalveolar lavage on infection control in patients with severe ventilator-associated pneumonia

**DOI:** 10.1016/j.clinsp.2025.100699

**Published:** 2025-06-11

**Authors:** Chunfeng Sheng, Xun Xu, Xiaobo Song, Bangfeng Zhao, Yu Zhang, Fengli Si

**Affiliations:** Department of Respiratory and Critical Medicine, Songjiang Hospital Affiliated to Shanghai Jiao Tong University School of Medicine, Shanghai, China

**Keywords:** Fiberoptic bronchoscopy, Bronchoalveolar lavage, Ventilator-associated pneumonia, Infection control, Respiratory mechanics

## Abstract

**Objectives:**

To evaluate the impact of Fiberoptic Bronchoscopy (FOB) with Bronchoalveolar Lavage (BAL) on infection control in patients with severe Ventilator-Associated Pneumonia (VAP) and compare it with conventional treatments to provide a reliable basis for clinical therapy.

**Method:**

This retrospective study included 86 patients with severe VAP who were admitted to the hospital between November 2019 and June 2023. Based on the treatment regimen, the patients were divided into two groups: a control group (conventional lavage and suction therapy, *n* = 32) and an observation group (FOB with BAL, *n* = 54).

**Results:**

The bacterial clearance rate of the observation group was significantly higher than in the control group (87.93 % vs. 70.27 %, *p* < 0.05). On days 5 and 7 post-treatment, the CPIS scores of the observation group were markedly lower than those of the control group (*p* < 0.05). After Post-treatment, the observation group demonstrated significantly superior respiratory mechanics (PIP, Cdyn, Raw), blood gas analysis parameters (PaO_2_, PaO_2_/FiO_2_, PaCO_2_), and pulmonary function indicators (PEF, PEEPi) compared to the control group (*p* < 0.05). The levels of inflammatory markers in the observation group were notably reduced (*p* < 0.05). Additionally, the duration of mechanical ventilation, infection control, and length of stay were significantly shorter in the observation group (*p* < 0.05). There was no significant difference in the incidence of complications between the two groups (*p* > 0.05).

**Conclusions:**

FOB with BAL as adjunctive therapy for VAP demonstrated significant advantages in enhancing respiratory mechanics, pulmonary function, blood gas analysis, inflammatory response, and bacterial clearance rate while effectively reducing mechanical ventilation duration and length of stay.

## Highlights

• FOB with BAL enhances infection control and improves overall patient prognosis.
• FOB with BAL provides a clear basis for pathogen diagnosis.
• FOB with BAL aids in enhancing the effectiveness of antibiotic therapy.
• FOB with BAL is safe for use in severe VAP patients.

## Introduction

Ventilator-Associated Pneumonia (VAP) is a prevalent and severe nosocomial infection in the ICU,^1^^,^^2^ typically occurring between 48 h after mechanical ventilation and 48 h post-extubation. Its incidence can reach 42 % with a mortality rate of 50 %.^3^ The occurrence of VAP is closely associated with prolonged mechanical ventilation time, extended hospital stay, and multidrug-resistant bacteria infections,^4^^,^^5^ leading to difficulties in infection control and suboptimal treatment outcomes. The current foundational treatment relies mainly on sensitive antibiotics combined with sputum suction therapy.^6^^,^^7^ This approach aims to enhance ventilation by suppressing persistent pulmonary inflammation. However, due to the limitations of sputum suction therapy in clearing airway secretions, especially when the secretions are thick, or the patient’s expectoration function is weakened, therapeutic outcomes often fall short of expectations.

With advancements in medical technology, Fiberoptic Bronchoscopy (FOB)^8^^,^^9^ and Bronchoalveolar Lavage (BAL)^10^^,^^11^ have gradually assumed pivotal roles as adjunctive therapeutic modalities in treating VAP. Through precise localization via bronchoscopy, BAL provides etiological evidence to aid in diagnosing infections and clears pathogenic microorganisms, secretions, and debris from the airways, alleviating the infection burden and addressing the shortcomings of conventional suction treatments. Although BAL has shown some efficacy in improving lung infections, shortening mechanical ventilation time, and reducing hospital stays, its effect on reducing complications and mortality remains controversial. Therefore, this study aimed to further evaluate the specific impact of FOB with BAL on infection control in patients with severe VAP to provide a more reliable basis for clinical treatment.

## Materials and methods

### Baseline data

This retrospective study collected clinical data from 86 patients with severe VAP who were hospitalized in the hospital between November 2019 and June 2023 and met the predefined inclusion and exclusion criteria. Based on the annual incidence rate of VAP in the hospital, historical data indicate that approximately 12.5 % of mechanically ventilated patients develop VAP, corresponding to roughly 25 cases per year. The number of cases included in this study reflects the actual clinical population during the study period and is thus representative, constituting a reasonable sample size for retrospective analysis. Patients were divided into groups based on different treatment regimens: the control group (*n* = 32) received conventional lavage and suction therapy, whereas the observation group (*n* = 54) received FOB with BAL and suction therapy.

### Inclusion criteria

(1) Age ≥ 18-years; (2) Meeting the diagnostic criteria for severe VAP as outlined in the Guidelines for the Diagnosis, Prevention, and Treatment of Ventilator-Associated Pneumonia (2013), with a definitive diagnosis based on clinical manifestations, imaging findings (CT or X-Ray), and laboratory tests; (3) Duration of mechanical ventilation exceeding 48 h, with pulmonary infection symptoms emerging within 48 h post-extubation; (4) Meeting at least one of the following clinical criteria: An increase in body temperature exceeding 1 °C or an absolute temperature > 38 °C; White blood cell count > 10 × 10⁹/L, or an elevation exceeding 25 % from baseline; Purulent respiratory secretions; Imaging evidence of newly developed or progressive pulmonary infiltrates.

### Exclusion criteria

(1) Patients with severe chronic liver disease (Child-Pugh class C), unstable coronary heart disease, intermittent myocardial infarction, or acute and chronic hepatic or renal failure; (2) Individuals with psychiatric or psychological disorders that impaired cooperation; (3) Patients with atelectasis, severe pulmonary hypertension, tuberculosis, lung cancer, or other pulmonary conditions that may affect study outcomes; (4) Individuals with contraindications to FOB; (5) Pregnant or lactating women; (6) Patients with concurrent infectious diseases; (7) Patients who were transferred to another hospital, withdrew, or died during the study period.

This study was approved by the Ethics Committee of Songjiang Hospital Affiliated with Shanghai Jiao Tong University School of Medicine (Approval n° 201,608). This observational study was reported in accordance with the STROBE guidelines. The requirement for informed consent was waived by the Ethics Committee due to the retrospective nature of the study.

### Data collection

This study retrospectively analyzed the electronic medical records of the patients, including medical orders, nursing notes, laboratory test results, and imaging reports. The primary observational indicators include: (1) Basic characteristics: sex, age, Body Mass Index (BMI), length of hospital stay, duration of Mechanical Ventilation (IMV), tracheostomy records, and discharge outcomes; (2) Bronchoscopy: the specific timing, frequency, and average rate of procedures were recorded; (3) Laboratory results: 3–5 mL fasting venous and arterial blood samples were collected from each patient on the day before and the day after BAL, respectively, and these samples were used for blood routine examination, gas analyses, inflammatory markers, and biochemical indicators using hospital-specific equipment. All data were meticulously recorded and organized by trained medical personnel.

### Treatment methods

After hospitalization, all patients received antibiotic therapy with adjustments based on susceptibility test results to ensure the administration of sensitive antibiotics.

The control group received routine lavage and suction therapy, whereas the observation group received psychological reassurance to alleviate anxiety prior to FOB with BAL. Additionally, patients were intravenously administered diazepam (15 mg) for sedation and 2 % lidocaine for local pharyngeal anesthesia. The bronchoscope was inserted nasally, and the patient was placed in a supine position. Vital signs, including blood pressure, heart rate, and oxygen saturation, were continuously monitored using a GE Solar 8000 multifunctional monitor. Simultaneously, an independent respiratory mechanics monitoring system was employed to dynamically assess Peak Inspiratory Pressure (PIP), Dynamic Compliance (Cdyn), Airway Resistance (Raw), and Work of Breathing (WOB).

After precisely identifying the pulmonary lesion area, 37 °C sterilized 0.9 % saline was slowly infused through the biopsy port, with a volume of 20 mL for each infusion, accumulating to a total BAL volume of 100 mL. After each infusion, the solution was retained within the alveoli for approximately 2 min to ensure thorough contact. Subsequently, a negative pressure suction device was used to collect the BAL fluid and secretions, with the suction pressure maintained at −100 to −150 mmHg. The collected BAL fluid and secretions were used for bacterial culture and antimicrobial susceptibility testing. Upon completion of the BAL, 20 mL of a diluted solution of antibiotic was instilled based on the susceptibility results, with a portion of the sample retained for documentation or further analysis. Treatment was administered once every two days.

### Observation indicators

#### Strain culture and clearance rate

All testing procedures complied with relevant regulations, ensuring the operations were conducted in a sterile environment. Sputum samples were collected using single-use suction tubes, primarily from the lower respiratory tract, and were subjected to bacterial culture and antimicrobial susceptibility testing. The types of cultured strains were recorded, and bacterial clearance rates were calculated and compared.

### Evaluation of clinical infection conditions

Changes in CPIS scores were recorded and analyzed between the two groups. Scoring included seven relevant indicators, such as body temperature, white blood cell count, and tracheal secretions. The score ranges from 0 to 12, and antibiotic treatment may be discontinued if the score is <6.

### Respiratory mechanics and pulmonary function parameters

A respiratory monitoring system was used to assess PIP, Cdyn, and Raw before and after each lavage, and a pulmonary function analyzer was used to measure pre- and post-treatment PEF and PEEPi levels.

### Hospitalization

Data on mechanical ventilation time, infection control time, and length of stay were collected from the two groups, and the differences between the three indicators were compared.

### Biochemical index assessment

An arterial blood gas analyzer was used to determine the PaO_2_, PaO_2_/FiO_2_, and PaCO_2_ levels. An enzyme-linked immunosorbent assay was used to measure PCT, CRP, and IL-6 levels before and after treatment.

### Occurrence of complications

The complications, including low oxygen saturation, irritating cough, and sinus bradycardia, were recorded and compared between the two groups.

### Statistical analysis

Statistical analyses were conducted using SPSS 15.0, while statistical graphs were generated using GraphPad Prism 20.0. The normality of all continuous variables was assessed using the Shapiro-Wilk test, confirming that the data followed a normal distribution. Accordingly, continuous data were expressed as mean ± Standard Deviation (mean ± SD) and analyzed using the *t*-test. Categorical variables, such as sex, incidence of complications, and adverse reaction grades, were described as frequency (n) and percentage (%). Intergroup comparisons were conducted using the Chi-Square test or Fisher's exact test. All tests were considered statistically significant at *p* < 0.05.

## Results

### Comparison of baseline data

The results of the baseline data comparison were similar between the two groups (*p* > 0.05) (Table 1).

**Table 1:** Comparison of baseline data between the two groups.

Group	n	Sex (M/F)	Mean age (years)	BMI (kg/m^2^)	APACHEII score (points)	Disease type	
						Early-onset	Late-onset
Control group	32	19/13	59.6 ± 9.2	24.79 ± 3.22	19.45 ± 2.88	19	13
Observation group	54	32/22	61.9 ± 8.7	23.81 ± 4.53	19.36 ± 2.57	26	28
*t*	‒	0.120	0.661	1.090	0.150	1.015	
*p*	‒	0.730	0.513	0.279	0.881	0.314	

### Comparison of strain culture status and bacterial clearance rates between the two groups

In the comparison of clearance rates, the observation group demonstrated a significantly higher clearance rate of 87.93 % (*p* < 0.05) (Table 2).

**Table 2:** Comparison of strain culture status and bacterial clearance rates between the two groups.

Group	n	Gram-negative bacillus				Gram-positive bacillus	Total	Bacteria clearance [case ( %)]
		Klebsiella	*Escherichia coli*	Haemophilus influenzae	Pseudomonas aeruginosa	Staphylococcus aureus		
Control group	32	4	13	2	13	5	37	26 (70.27)
Observation group	54	7	17	6	19	10	58	51 (87.93)
*χ*²	‒	‒	‒	‒	‒	‒	‒	4.588
*p*	‒	‒	‒	‒	‒	‒	‒	0.032

### Comparison of changes in CPIS score between the two groups

The CPIS scores of both groups on days 5 and 7 of treatment were significantly lower than before treatment (*p* < 0.05). There were also statistically significant differences in the CPIS scores between the two groups on days 5 and 7 (*p* < 0.05) (Table 3).

**Table 3:** Comparison of changes in CPIS score between the two groups.

Group	n	Before treatment	On the 5th day of treatment	On the 7th day of treatment
Control group	32	8.93 ± 2.41	6.59 ± 1.37	5.49 ± 0.85
Observation group	54	8.55 ± 2.13	4.93 ± 1.22	2.95 ± 0.77
*t*	‒	0.120	0.661	1.090
*p*	‒	0.730	0.513	0.279

### Comparison of changes in respiratory mechanics between the two groups

**Fig. 1:**
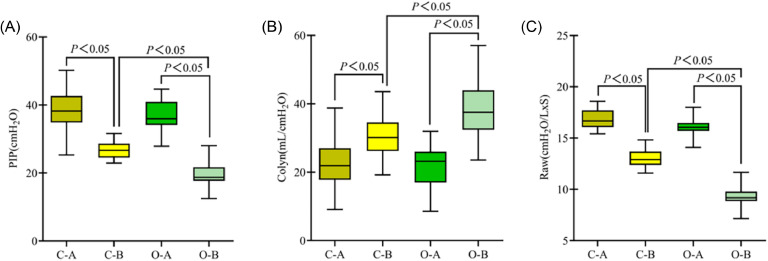
**Comparison of changes in respiratory mechanics between the two groups. (**A) PIP; (B) Cdyn; (C) Raw; (C-A) Control group before treatment; (C-B) control group after treatment; (O-A) Observation group before treatment; (O-B) Observation group after treatment; *p* < 0.05 indicates a statistically significant difference.

Respiratory mechanics in both groups showed no significant differences before treatment (*p* > 0.05). However, after treatment, both groups exhibited substantial improvements in respiratory mechanics, with the observation group demonstrating a more pronounced enhancement (*p* < 0.05) (Fig. 1).

### Comparison of hospitalization between the two groups

**Fig. 2:**
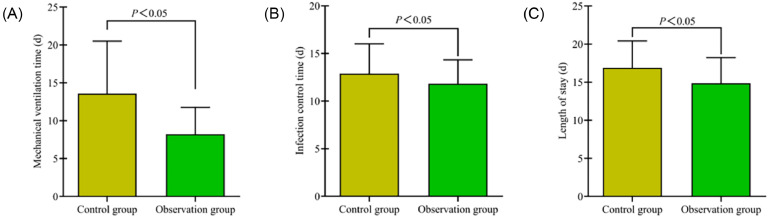
**Comparison of hospitalization between the two groups.** (A) Mechanical ventilation time; (B) Infection control time; (C) Length of stay; *p* < 0.05 indicates a statistically significant difference.

The hospitalization indices were significantly lower in the observation group than in the control group (*p* < 0.05) (Fig. 2).

### Comparison of blood gas analysis indices between the two groups

**Fig. 3:**
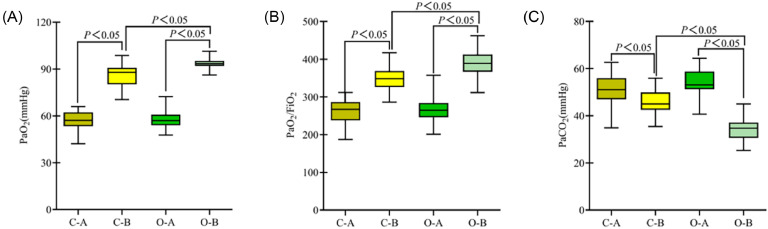
**Comparison of blood gas analysis indices between the two groups.** (A) PaO_2_; (B) PaO_2_/FiO_2_; (C) PaCO_2_; (C-A) Control group before treatment; (C-B) Observation group before treatment; (O-B) Observation group after treatment; *p* < 0.05 indicates a statistically significant difference.

The blood gas indices of the two groups were similar before treatment (*p* > 0.05). Both groups showed significant improvement after treatment, with the observation group demonstrating a more pronounced improvement (*p* < 0.05) (Fig. 3).

### Comparison of inflammatory markers between the two groups

**Fig. 4:**
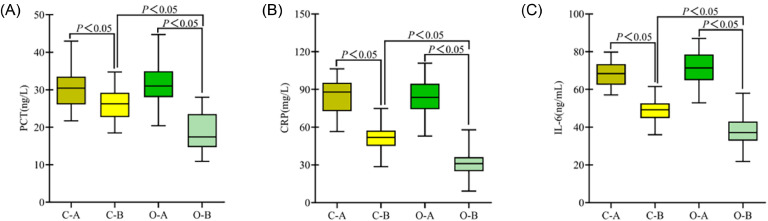
**Comparison of inflammatory markers between the two groups.** (A) PCT; (B) CRP; (C) IL-6; (C-A) Control group before treatment; (C-B) Control group after treatment; (O-A) Observation group before treatment; (O-B) Observation group after treatment; *p* < 0.05 indicates a statistically significant difference.

Before treatment, the levels of inflammatory markers were similar between the two groups (*p* > 0.05). After treatment, the levels of inflammatory markers significantly decreased in both groups and were lower in the observation group (*p* < 0.05) (Fig. 4).

### Comparison of pulmonary function between the two groups

Pulmonary function did not significantly differ between the two groups before treatment (*p* > 0.05). Both groups showed significant improvement in pulmonary function after treatment, with the observation group demonstrating a more pronounced improvement (*p* < 0.05) (Table 4).

**Table 4:** Comparison of pulmonary function between the two groups.

Group	n	PEF (L/s)		PEEPi (mmHg)	
		Before treatment	After treatment	Before treatment	After treatment
Control group	32	83.73 ± 14.27	127.05 ± 26.81	0.94 ± 0.24	0.63 ± 0.18
Observation group	54	85.29 ± 15.79	157.22 ± 31.88	0.97 ± 0.34	0.51 ± 0.14
*t*	‒	0.459	4.492	0.438	3.449
*p*	‒	0.648	<0.001	0.662	0.001

### Comparison of complications between the two groups

The incidence of complications between the two groups was not significantly different (*p* > 0.05) (Table 5). The symptoms were relieved after symptomatic treatment with oxygen and nebulization.

**Table 5:** Comparison of complications between the two groups.

Group	n	Low oxygen saturation	Irritating cough	Sinus bradycardia	Total
Control group	32	1	0	1	6.25 % (2/32)
Observation group	54	1	2	1	7.41 % (4/54)
*OR*	‒	‒	‒	‒	0.83
a*p*	‒	‒	‒	‒	1.000

a Fisher's exact test.

## Discussion

This study compared the clinical outcomes of two distinct treatments for severe VAP. By evaluating improvements in respiratory mechanics, blood gas analysis, and other factors, FOB with BAL demonstrated a more pronounced advantage. These results suggest that FOB with BAL as an adjunctive therapy for VAP can significantly enhance infection control and improve overall patient prognosis.

The treatment of severe VAP, primarily caused by Gram-negative bacilli, is challenging. The efficacy of conventional antimicrobial agents is often inadequate when used alone, and the issue of drug resistance progressively worsens.^11^^,^^12^ The primary pathological mechanism involves a severe inflammatory response in the respiratory tract, alveoli, and pulmonary interstitium, accompanied by a substantial accumulation of purulent and viscous secretions that severely impair pulmonary ventilation and gas exchange. Moreover, a persistent inflammatory response may further induce atelectasis or exacerbate pulmonary consolidation, ultimately triggering or aggravating respiratory failure.^13^ Therefore, the timely and effective clearance of respiratory secretions, attenuation of airway inflammation, and restoration of alveolar ventilation are critical therapeutic interventions for these patients. FOB, combined with BAL, has emerged as an integrated diagnostic and therapeutic modality providing a novel treatment strategy for patients with severe pneumonia. This technique enables precise bronchoscopic access to the lesion regions, facilitating repeated BAL to remove inflammatory secretions and necrotic tissues from the alveoli, thereby rapidly improving the ventilation function.^14^ Simultaneously, it allows the collection of high-quality specimens during BAL for pathogen culture, guides targeted antibiotic therapy, and enhances treatment efficacy and precision.^15^^,^^16^ The results of this study indicated that the bacterial clearance rate in the observation group was significantly higher than that in the control group and that the CPIS scores on days 5 and 7 post-treatment in the observation group were markedly lower than those in the control group. This suggests that FOB with BAL provides a clear basis for pathogen diagnosis and effectively removes pathogens and secretions from the alveoli, thereby enhancing the efficacy of anti-infective treatment. FOB with BAL aids in enhancing the effectiveness of antibiotic therapy by eradicating the infection site and reducing the bacterial load because of the severe infection risks and issues of pathogen resistance typically faced by patients with VAP.

Furthermore, the results indicated that the respiratory mechanical parameters (PIP, Cdyn, and Raw), blood gas analysis indicators (PaO_2_, PaO_2_/FiO_2_, and PaCO_2_), and pulmonary function indices (PEF and PEEPi) in the observation group were significantly superior to those in the control group, demonstrating that this technique can effectively improve the ventilation status and enhance pulmonary function in patients. BAL thoroughly irrigates the lesion area, not only diluting and suctioning viscous inflammatory secretions to alleviate airway obstruction effectively but also stimulating and enhancing the patient's cough reflex, facilitating the discharge of deep sputum and further clearing residual secretions within the bronchi and alveoli. This process aids in reconstructing a patent airway environment and improves alveolar ventilation and gas exchange, thereby enhancing pulmonary function. Improving pulmonary function not only serves as a direct manifestation of inflammation control but is also closely linked to a patient's oxygenation status, degree of dyspnea, and clinical prognosis. In patients with severe pneumonia complicated by respiratory failure, pulmonary function recovery is a pivotal determinant of ventilator weaning, hospitalization duration reduction, and mortality rate reduction.^17^^,^^18^ Therefore, adjuvant therapy with BAL to enhance airway drainage and restore ventilatory function has significant clinical value.

Inflammatory factors play a pivotal role in the onset and progression of severe pneumonia and respiratory failure. Inadequate regulation of the inflammatory response may trigger Systemic Inflammatory Response Syndrome (SIRS) and exacerbate localized pulmonary inflammation, markedly increasing pulmonary and airway secretions. Consequently, this exacerbates ventilatory dysfunction, induces persistent alveolar structural damage, and accelerates disease progression.^19^^,^^20^ In this study, the post-treatment levels of PCT, CRP, and IL-6 in the observation group were significantly lower than those in the control group (*p* < 0.05), suggesting that FOB combined with BAL therapy helps to reduce systemic inflammatory factor levels. FOB with BAL, an intervention performed under direct visualization with a fiberoptic bronchoscope, accurately targeted the lesion site and promptly removed purulent viscous secretions from the deep airways and alveoli. Repeated BAL not only improves the local microenvironment and inhibits the proliferation of pathogenic microorganisms but also facilitates the collection of high-quality secretions for pathogen culture, thereby guiding the selection of individualized targeted antimicrobial agents. Post-lavage antimicrobial therapy can act directly on the lesion area, increase the local drug concentration, and enhance the antimicrobial clearance effect, thereby effectively controlling the inflammatory response and promoting the recovery of pulmonary function.

Although FOB combined with BAL is somewhat invasive and may pose potential risks such as hypoxemia, bronchospasm, and arrhythmias in patients with severe VAP, numerous studies have confirmed that under rigorous monitoring, appropriate sedation, and proper airway management, the overall safety of the procedure remains high. During the BAL procedure conducted under mechanical ventilation, optimal control of the BAL volume and suction pressure, along with close monitoring of blood oxygen saturation and vital signs, can significantly minimize the incidence of complications. The results of this study showed that the length of stay, infection control time, and mechanical ventilation time of the observation group were significantly shorter than those of the control group, indicating that the treatment plan for the observation group was more effective in controlling infections and promoting pulmonary function recovery, reducing the dependency of patients on mechanical ventilation. Although there was no statistically significant difference in the incidence of complications between the two patient groups, the overall complication rate was relatively low, indicating that FOB with BAL is safe for use in patients with severe VAP. Although some patients experienced adverse reactions such as decreased oxygen saturation, irritating cough, and sinus bradycardia, these were alleviated with appropriate symptomatic treatment. Therefore, in patients with severe VAP, based on a careful assessment of benefits and risks, the rational implementation of FOB combined with BAL not only facilitates early identification of the etiology and improvement in pulmonary function but also provides a foundation for individualized antimicrobial therapy, demonstrating significant clinical applicability and promising prospects for broader adoption.

This study had certain limitations that warrant attention. First, this was a single-center retrospective study with a relatively small sample size, which may constrain the generalizability and representativeness of the findings. Future research based on a multicenter, large-scale, prospective study design would be beneficial to further validate these results’ reliability and clinical applicability. Furthermore, while this study used the definition of VAP for analysis, the more widely used classification system for Ventilator-Associated Events (VAE) in recent years may offer greater sensitivity and practical utility in clinical practice. Therefore, future studies should consider incorporating VAE metrics to enhance this study’s accuracy and real-world relevance.

## Conclusion

In summary, FOB with BAL, as an adjunctive therapy for VAP, significantly improves respiratory mechanics, pulmonary function, blood gas analysis, and inflammatory response, shortens mechanical ventilation duration and length of stay, and markedly enhances bacterial clearance rate. These results strongly support the efficacy of VAP treatment.

## Authors’ contributions

Chunfeng Sheng and Fengli Si conceived the study and designed the experiments. Xun Xu and Xiaobo Song contributed to the data collection. Bangfeng Zhao and Yu Zhang performed the data analysis and interpreted the results. Chunfeng Sheng wrote the manuscript. Fengli Si contributed to the critical revision of article. All authors read and approved the final manuscript.

## Funding

No funding was received for conducting this study.

## Declaration of competing interest

The authors declare no conflicts of interest.
